# A Novel Interaction Between the TLR7 and a Colchicine Derivative Revealed Through a Computational and Experimental Study

**DOI:** 10.3390/ph11010022

**Published:** 2018-02-13

**Authors:** Francesco Gentile, Marco A. Deriu, Khaled H. Barakat, Andrea Danani, Jack A. Tuszynski

**Affiliations:** 1 Department of Physics, University of Alberta, AB T6G 2E1 Edmonton , Canada; fgentile@ualberta.ca; 2 Istituto Dalle Molle di Studi Sull’intelligenza Artificiale (IDSIA), Scuola Universitaria Professionale della Svizzera Italiana (SUPSI), Università della Svizzera Italiana (USI), CH-6928 Manno, Switzerland; deriu.marco@gmail.com (M.A.D.); andrea.danani@gmail.com (A.D.); 3 Faculty of Pharmacy and Pharmaceutical Sciences, University of Alberta, AB T6G 2H1 Edmonton, Canada; kbarakat@ualberta.ca; 4 Department of Mechanical and Aerospace Engineering, Politecnico di Torino, 10129 Torino, Italy; 5 Department of Oncology, University of Alberta, AB T6G 1Z2 Edmonton, Canada

**Keywords:** TLR7, colchicine, imiquimod, innate immune system, off-target interaction

## Abstract

The Toll-Like Receptor 7 (TLR7) is an endosomal membrane receptor involved in the innate immune system response. Its best-known small molecule activators are imidazoquinoline derivatives such as imiquimod (R-837) and resiquimod (R-848). Recently, an interaction between R-837 and the colchicine binding site of tubulin was reported. To investigate the possibility of an interaction between structural analogues of colchicine and the TLR7, a recent computational model for the dimeric form of the TLR7 receptor was used to determine a possible interaction with a colchicine derivative called CR42-24, active as a tubulin polymerization inhibitor. The estimated values of the binding energy of this molecule with respect to the TLR7 receptor were comparable to the energies of known binders as reported in a previous study. The binding to the TLR7 was further assessed by introducing genetic transformations in the TLR7 gene in cancer cell lines and exposing them to the compound. A negative shift of the IC_50_ value in terms of cell growth was observed in cell lines carrying the mutated TLR7 gene. The reported study suggests a possible interaction between TLR7 and a colchicine derivative, which can be explored for rational design of new drugs acting on this receptor by using a colchicine scaffold for additional modifications.

## 1. Introduction

In humans, toll-like receptors (TLRs) are a family of ten receptors (TLR1-10), which are part of the innate immune system. The innate immune system’s main function is to recognize pathogen-associated molecular patterns (PAMPs), which belong to microbial pathogens such as bacteria, viruses, fungi and protozoa, in addition to damage-associated molecular patterns (DAMPs) coming from damaged cells [1,2]. It is commonly assumed that each TLR receptor recognizes specific molecular patterns, and all of them are multi-domain, trans-membrane proteins localized in the cellular membrane, except for TLR7, 8 and 9 which are found on the endosomal membrane [1]. The common structure of TLRs involves an ectodomain comprising leucine-rich repeats (LRR), a transmembrane domain and a Toll/IL-1 receptor (TIR) domain, which initiates the cascade of signaling after a PAMP/DAMP recognition event by engaging TIR domain-containing proteins such as myeloid differentiation primary response 88 (MyD88) and TIR-domain-containing adapter- inducing interferon-β (TRIF) proteins. This transduction pathway results in the production of cytokines, chemokines and type I interferons to protect the cells from microbial invasion [2]. 

**Figure 1:**
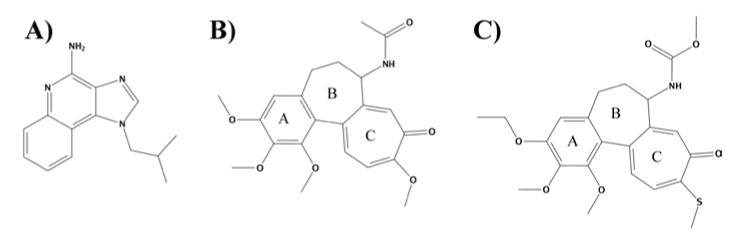
Chemical structure of **A**) R-837, **B**) colchicine and **C**) CR42-24. For the latter two, the rings are named according to the convention used in the article (A, B, C).

The TLR7 is an endosomal transmembrane protein composed of 1049 amino acid residues, divided between an endosomal ectodomain with twenty-seven LLR arranged in a horseshoe structure, a trans-membrane domain and a TIR domain located in the cellular compartment. The functional form of TLR7 is a homodimer complex [3]. The natural ligands recognized by TLR7 are viral single-strand RNA (ssRNA). The signaling cascade is mediated through the interaction with MyD88 [4]. Synthetic ligands that bind to TLR7 are synthetic ssRNA and nucleoside analogues, such as imidazoquinoline, adenosine and guanosine derivatives [5,6]. Recently, different TLR7-targeting small molecules were developed as immune response modifiers for anti-cancer and anti-viral therapy. Imiquimod (R-837) (see Figure 1A) was the first Food and Drug Administration (FDA)-approved TLR7 agonist, and its formulation containing 5% of cream (Aldara®) is used to treat genital warts caused by human Papilloma virus (HPV) infection, and malignant skin cancers. This imidazoquinoline derivative induces the production of pro-inflammatory cytokines and other molecules upon TLR7 activation [7,8]. Resiquimod (R-848) and CL097 are two dual TLR7/8 agonists with a similar effect on R-837 [8,9,10]. Interestingly, efforts were made for developing both activators and inhibitors of TLR7. While activation of the receptor is sought to enhance the immune response to viral agents, its inhibition gains interest in the treatment of autoimmune disease such as lupus [11]. In the context of cancer treatment, again the interest is in both directions. First, TLR7 activation is an assessed strategy for cancer immunotherapy, where its stimulation leads to the secretion of anti-tumor cytokines [12,13,14]. However, it has been reported that the receptor expression is upregulated in human pancreatic tumor cells, its signaling regulates carcinogenesis, TLR7 ligation promotes tumor progression and TLR7 blockade protects against cancer development [15]. This suggests a possible role of TLR7 inhibitors in the treatment of pancreatic cancer [16].

Colchicine is a natural compound extracted from the meadow saffron (*Colchicum autumnale*). It was identified as the first microtubules-destabilizing agent, and it is an FDA-approved drug for the treatment of familial Mediterranean fever and gout. Colchicine acts by binding to a binding site in the β-tubulin monomer, resulting in a curved tubulin dimer conformation, which prevents microtubule assembly, thus inhibiting mitosis. Although it has a cytotoxic effect against cancer cells due to their accelerated rate of mitosis, the use of colchicine in chemotherapy is limited by high toxicity affecting normal cells and multidrug resistance [17]. To overcome these problems, numerous colchicine derivatives were synthesized and tested in the past few years, mainly by modifying the B and C rings of the original structure (Figure 1B) [18]. Through an iterative process of computational screening and subsequent cytotoxicity testing the Tuszynski lab identified a number of compounds that showed enhanced binding affinity for βIII tubulin. A compound coded CR42-24 was selected based on its high affinity to bind βIII tubulin, and showing improved cytotoxicity over other derivatives. CR42-24 is a colchicine analogue with the following modifications of the original structure (see Figure 1C): one of the methoxy groups of the A ring is replaced by an ethoxy group, the acetamide in B ring is replaced by a methyl carbamate, and the methoxy group of the C ring is substituted by a methyl sulfanyl group. CR42-24 had shown great promise in its ability to treat cancer and it had been shown to be highly effective against breast cancer, leukemia, and lung cancer cell lines. An extensive review of this compound and a number of similar structures has been recently published where detailed information about their profiles can be found [19]. 

Recently, it has been reported for the first time that R-837 might bind to the colchicine-binding site of tubulin, hence inhibiting tubulin polymerization [20]. Molecular docking of R-837 to this site revealed a binding mode similar to the experimentally obtained colchicine one, showing agreement with the colchicine site pharmacophores published earlier for active compounds [21]. In this research, we explored the reverse process, i.e., we aimed to understand if compounds binding to the colchicine binding site in tubulin could possibly bind to the R-837 binding site at the interface between the two TLR7 monomers. Starting from our recent computational model of the human TLR7 model [22], we employed a series of *in silico* methods in order to identify and quantitatively characterize the binding mode of CR42-24 to the TLR7 site. We also employed a cell line-based experiment to demonstrate the possible interaction between the compound and TLR7, by studying the effect that CR-24-42 has on cancer cells carrying either a wild-type or a mutant TLR7 gene, respectively.

CR42-24 is an in-house discovered and developed, potent microtubules-destabilizing agent with promising anti-cancer activities. The results reported in this article are of potential interest not only to predict possible side effects of CR42-24 on the immune system, but also to enable the design of novel small molecules targeting TLR7 with structures based on this scaffold.

## 2. Results and discussion

### 2.1. Homology Modeling

**Figure 2:**
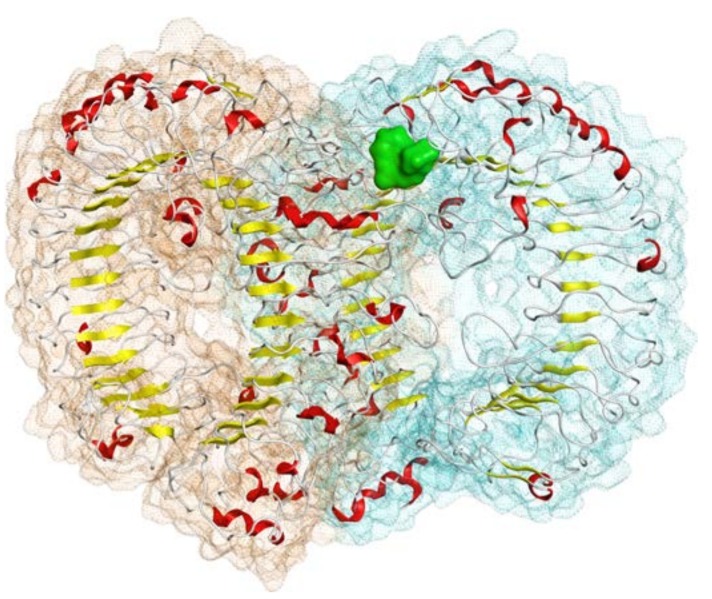
Structure of the homodimeric model of the TLR7 ectodomains obtained in our previous work from the TLR8 template. The a and b monomer surfaces are represented in brown and cyan, respectively. Each monomer secondary structures are colored as follow: helices in red, strands in yellow, loops and turns in white. The R-837 binding site targeted in this study, lying at the interface of the two monomers, is represented as green surface.

We have already reported the validation results for our TLR7 model in [22]. To summarize, the sequence identity between the TLR7 and TLR8 ectodomains was around 46%. The model that was generated includes 1514 residues equally divided in two monomers (757 residues each, hereinafter referred to as monomer a and b, respectively), excluding the residues that are removed after the proteolytic cleavage required for TLR7 activation [23]. Our model showed ERRAT scores and dihedral angle values (calculated with PROCHECK) comparable with the experimental template and the other TLR7 homology models reported in literature [24,25,26], especially after the minimization step. In fact, just 1% of modeled residues showed dihedral values in the disallowed zone of the Ramachandran plot. The 3D structure of our TLR7 model is reported in Figure 2.

### 2.2. Molecular Docking

**Figure 3:**
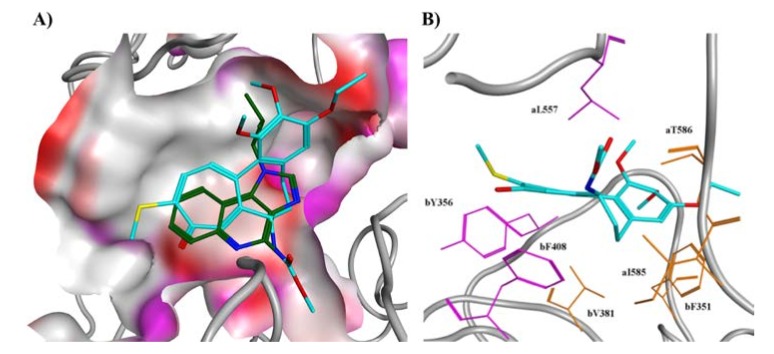
Analysis of the predicted binding pose of CR42-24 to the R-837 binding site of TLR7. **A**) Comparison between the docking poses of R-837 (dark green carbons) and CR42-24 (cyan carbons) to the TLR7 binding site. Red surface indicates solvent-exposed zones, white surface indicate hydrophobic zones and purple surface indicates polar zones of the site. Key common interactions are the position of the three-ring moieties in the hydrophobic zone of the binding pocket and the orientation of amine groups in both the compounds towards the polar zone surrounding aD555. In addition, the carbonyl group of the C ring of CR42-24 was oriented in proximity of bK432. **B**) Hydrophobic contacts of CR42-24 with the TLR7 residues of the binding pocket. From the reported docking pose, we observed two distinct hydrophobic zones interacting with the compound, one constituted by aL557, bY356 and bF408 (represented in purple) and the other one by aI585, aT586, bF351 and bV381 (represented in orange). Two hydrophobic clefts, interacting with the same compound rings (A and C) as in our model, were observed for colchicine bound to the tubulin site. The pose is represented in an orientation similar to Figure S6 from [20] for comparison with the colchicine binding pose to the tubulin site.

Among the top five poses obtained from the docking simulations, we identified one which showed a binding geometry similar to the R-837 pose obtained and validated in our previous study [22] (Figure 3A). The remaining four poses are reported in Figure S1. The binding energy of the selected pose, calculated with the Generalized Born Volume Integral/Weighted Surface Area (GBVI/WSA) scoring function [27], was −8.194 kcal/mol. The binding energy of R-837, calculated by rescoring the docking pose with the GBVI/WSA method, was −4.991 kcal/mol. Regarding the specific interactions with the residues of the binding pocket, R-837 was involved in hydrophobic contacts mainly with residue aL557 and bF408 (where a and b refer to the first and second monomer, respectively), having the three-ring structure interposed between the two hydrophobic residues. The same contacts, in addition to others, were observed for the three rings of CR42-24, although the higher flexibility of the B ring resulted in a less planar geometry, when compared with R-837. R-837 also showed hydrogen bonding with the charged side chain of aD555 and the backbone oxygen of aT586. Although we did not observe any hydrogen bonds being established in the CR42-24-TLR7 complex, the amine of the acetamide group of the compound was positioned in the same zone of the amine group of R-837, a polar-favorable site of the binding pocket surrounding the negatively charged side chain of residue aD555. Also, the carbonyl group of the C ring of CR42-24 was positioned in a polar-favorable zone lying around the positively charged side chain of residue bK432 

Finally, we observed some similarities between the docking pose of CR42-24 and the colchicine conformation co-crystallized in the tubulin binding site as reported by Courbet et al [20], especially in terms of hydrophobic contacts. Indeed, in tubulin, the C ring of colchicine interacted through van der Waals interactions with a first hydrophobic pocket composed by αV181, αS178, and βV315, where α and β indicate the respective tubulin monomer. In our TLR7-bound model of CR42-24, the corresponding C ring was embedded in a hydrophobic cleft composed by aL557 and the aromatic rings of bY356 and bF408 (labelled in purple in Figure 3B). In tubulin, the A ring of colchicine was embedded in a second hydrophobic pocket, constituted by several residues. In TLR7, similarly, the corresponding A ring in CR42-24 is also embedded in a second hydrophobic zone, constituted by residues aI585, aT586, bF351 and bV381 (in orange in Figure 3B). The superposition between the CR42-24 docked pose in TLR7 and the N-deacetyl-N-(2-mercaptoacetyl)-colchicine (DAMA-colchicine) binding pose to tubulin found in the pdb structure 1SA0 [28] is reported in Figure S2.

### 2.3. Molecular Dynamics Simulations and MM/GBSA Calculations

**Figure 4:**
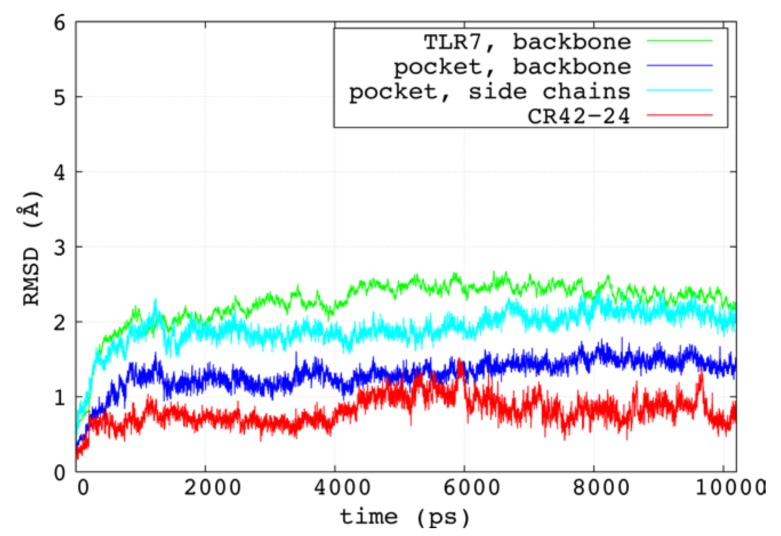
RMSD trends for the TLR7 backbone atoms (green), the backbone atoms of the binding pocket residues (blue), the side chain atoms of the binding pocket (cyan) and the heavy atoms of the CR42-24 molecule (green), calculated between the initial phase of restraint release and the end of the 10 ns-long production simulation (10.2 ns of total time).

Although the assessed power of docking to identify near-native poses of ligands within binding sites, some limitations arise when using these methods. Firstly, the binding site flexibility is at least fully neglected, although it is possible to introduce some degrees of flexibility for its side chains. Also, docking simulations provide a static picture of the binding interactions, and a binding energy calculated from on single pose rather than an average made over an ensemble. Lastly, the water molecules around the site are usually treated implicitly during docking. In the light of these considerations, we performed explicit solvent MD simulations of the ligand-receptor complex obtained from the docking. The root-mean square deviation (RMSD) trends of the protein backbone, the backbone and side chain atoms of the binding site and the heavy atoms of CR42-24 are reported in Figure 4. We observed RMSD values consistent with a stable, active ligand-receptor complex. Indeed, CR42-24 stabilized around 1 Å of RMSD value after about 4 ns of production simulation, and maintained the same fluctuation trend for the remaining simulated time. In our previous study, we observed a similar trend for the RMSDs of activators of TLR7, and consistently more unstable trends for inactive compounds in complex with the receptor [22]. The equilibrated RMSD trends of the protein backbone and the binding pocket were also similar to the trends observed in our earlier study. Stable RMSD trends were also observed when the average structure of the last 5 ns was used as reference (Figure S3).

The results of the molecular mechanics-generalized Born surface area (MM/GBSA) calculations are reported in Table 1. The total binding energies of the two complexes (TLR7 bound to R-837, from the previous study, and CR42-24) are comparable, with the CR42-24 complex showing a slightly more favorable energy. The highest differences observed in the single terms were for the van der Waals interactions (~11 kcal/mol less for the CR42-24 complex) and the polar solvation terms, calculated with the GB method. For these terms, the change in free energy for CR42-24 binding to the TLR7 was considerably larger (~14 kcal/mol more for the CR42-24 complex) than the R-837 one. This result suggests a binding of CR42-24 to the TLR7 dimer, which involves stronger hydrophobic interactions than the R-837 binding. Also, CR42-24 showed a more favorable energy value for the polar ligand-receptor interactions (~6 kcal/mol less than R-837). The two non-polar terms were comparable. 

**Figure 5:**
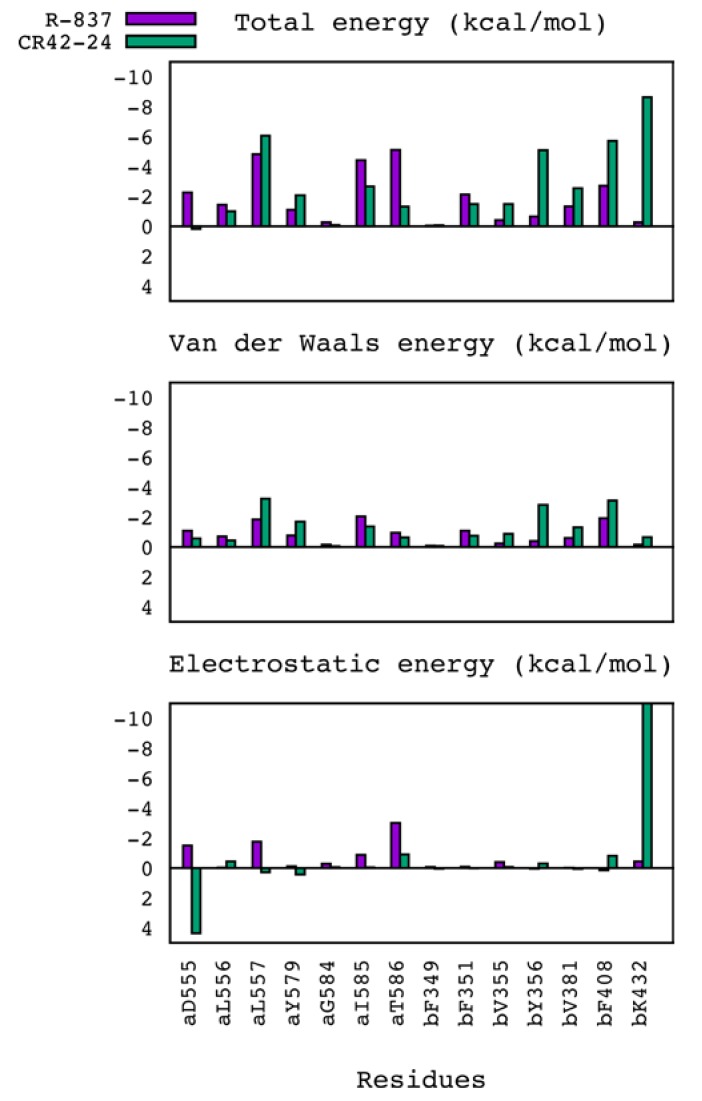
Decomposition of total, van der Waals and electrostatic interaction energies between the residues of the TLR7 pocket and the two compounds R-837 (violet) and CR42-24 (green). Similar patterns were observed for the total and van der Waals interactions of the two compounds with the TLR7 dimer, while the electrostatic interaction pattern was different. The unfavorable interaction of CR42-24 with the key residue aD555 was compensated by a favorable, electrostatic-driven interaction with bK432, which was not observed for other TLR7 ligands in our previous study.

In addition, we performed the decomposition of the MM/GBSA energies among the residues constituting the binding site of TLR7, in order to quantitatively assess the pattern of intermolecular interactions established between CR42-24 and the TLR7 dimer, and to compare it to the pattern observed for the R-837-TLR7 complex. The decompositions of the total binding energy between the residues within 5 Å of the docking poses, as well as the van der Waals and electrostatic terms, are reported in Figure 5. The residues aL557, aI585, bF351and bF408 showed the most similar contribution to the binding energies of the two compounds. Interestingly, these residues showed similar contributions also for the hydrophobic terms, whereas the electrostatic terms were either negligible or different between the two complexes. Also, CR42-24 showed a considerably more favorable total interaction energy with bY356 and bK432. For the first residue, the hydrophobic contribution was clearly more negative than the R-837 complex. Regarding bK432, we observed a very favorable electrostatic interaction energy for CR42-24 (~−11 kcal/mol), not present in the R-837-TLR7 complex. As already mentioned, this was due to a polar interaction involving the carbonyl group of the C ring of CR42-24, which established a stable, strong hydrogen bond with the side chain of bK432 during the simulation. This compensated the unfavorable total and electrostatic interactions between CR42-24 and aD555, which was reported as a key residue for the binding of imidazoquinoline and adenine derivatives to the TLR7 binding pocket [21], as in the case of R-837. By visually inspecting the MD production trajectory, this unfavorable contribution was observed to be due to a salt bridge established between aD555 and bK432, which locked the location of the former in close proximity to the carbonyl group of the CR42-24 C ring and far from the amine of the acetamide group (Figure S4).

### 2.4. Experiments

**Figure 6:**
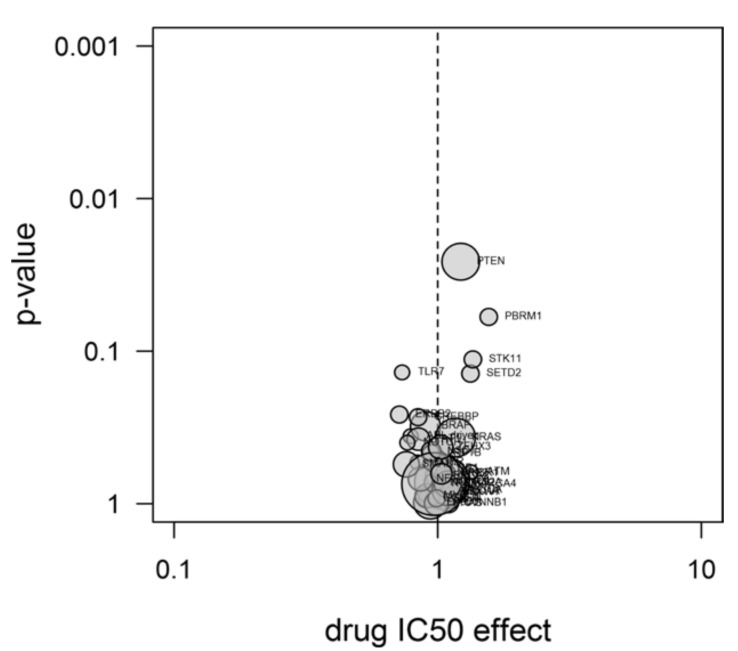
Volcano plot reporting the ANOVA analysis performed over thirty-nine cancer genes, including TLR7. The *x*-axis represents the factor of IC_50_ shift, while the y-axis reports the p-values, i.e., the confidence level for genetic association of mutations in a particular gene with a IC_50_ shift. The areas in the circles are proportional to the number of mutants present in the cell panel (each reported gene was present in at least three Oncolines^TM^ cell lines).

The results from the type II ANOVA test performed over the subset of cancer genes, including TLR7, are reported in the volcano plot in Figure 6. The cell lines carrying the mutated TLR7 gene were more sensitive to CR42-24, as it is clearly visible from the reported negative IC_50_ shift.

## 3. Materials and Methods

### 3.1. Model Building and Validation

The model for the endosomal domain of the human TLR7 heterodimer was the same described in our previous work [21]. Briefly, the x-ray structure of the human TLR8 dimer (Protein Data Bank [29] ID 3W3J) in complex with the TLR7/8 dual agonist CL097 [30] was selected as template for a homology modeling procedure in Molecular Operating Environment (MOE) 2013 [31]. *MOE-Align* [32] was used to align the two amino acid sequences. The modeling step was performed using the automated homology modeling tool of MOE, choosing the final model based on the Generalized Born Integration/Volume Integral (GB/VI) scoring function [27]. The Amber ff12SB force field parameters [33] were used for the protein. The two CL097 molecules present in the template were conserved as environment for the Induced Fit during the modeling protocol, assigning them the Extended Hückel Theory (EHT) parameters [34]. The protonation states of the model residues were assigned using *MOE Protonate3D* [35]. The ff12SB parameters were used for the protein, while the General Amber Force Field (GAFF) [36] parameters were assigned to the two ligand molecules using Antechamber [37]. An octahedral box of TIP3P water molecules [38] was built around the model, with a buffer of at least 12 Å between any complex’s atom and the edge of the box. The system was neutralized by adding Cl- ions. The TLR7 dimer was then minimized using the Amber *pmemd.cuda* engine [39]. The cutoff for long range interactions was set to 9 Å. Water molecule and ion positions were relaxed using a sequence of 1,000 steps of steepest descent and 1,000 steps of conjugate gradients minimization methods, keeping the protein and ligand atoms highly restrained through a harmonic potential with a force constant of 500 kcal/mol/Å^2^. Then a second minimization procedure was performed, with 2,000 and 3,000 steps of steepest descent and conjugate gradients methods, respectively. For this step, we restrained the protein backbone and the ligand heavy atoms only using a harmonic potential with a force constant of 4 kcal/mol/Å^2^. The optimized structure was then evaluated using the ERRAT [40] and PROCHECK [41] programs.

### 3.2. Ligand Preparation and Molecular Docking

The CR42-24 structure was prepared using the *LigPrep* module from Schrödinger’s Maestro [42], in order to obtain possible protonation states, tautomers and low-energy ring conformations at pH 5.5, which corresponds to the endosomal compartment value. The OPLS 2005 force field [43] was used for this process. 

The docking was performed using *MOE Dock*. The target structure was the R-837-TLR7 dimer complex obtained and validated in our previous work [22]. The choice of the target structure was made based on the evidence of a possible interaction of R-837 with the colchicine-binding site of tubulin [20], where CR42-24 is also likely to bind. The site to which performs the simulations was defined as the one occupied by the R-837 atoms, a cleft present at the interface of the two TLR7 monomers around residue aD555. The placement method was chosen as Triangle Matcher [44], returning thirty poses based on the London dG scoring [27]. The receptor was kept rigid for the refinement step, where five poses were retained based on the GBVI/WSA scoring function [27]. The five final poses were visually inspected.

### 3.3. Molecular Dynamics Simulations

The parameters required for the simulation of the small molecule were assigned from the General Amber Force Field (GAFF) [36] using Antechamber [37], whereas the parameters for the protein were chosen from the Amber ff12SB force field. Again, we inserted the system in an octahedral box of TIP3P water molecules, with at a minimum of 12 Å of buffer between any atom of the complex and the edge of the box. The system was neutralized by adding the correct number of Cl- ions. A value of 9 Å was set as cutoff for long range interactions. Using the *pmemd.cuda* engine, the system were initially minimized with the same minimization protocol used for the homology model. They were then heated up to 310 K in a time of 100 ps, using the Langevin thermostat with a time collision frequency of 2 ps and an integration time step of 0.5 fs. The target temperature was gradually reached after 150,000 steps and maintained for the last 50,000 of the heating protocol. A restraint of 2 kcal/mol/Å^2^ was imposed to the protein backbone and on the heavy atoms of the small molecule during this step, and constant volume conditions were applied. The restraints were slowly removed in four phases of 50 ps and constant pressure conditions, in each of which the force constant was reduced of 0.5 kcal/mol/Å^2^. The production NPT simulation was then run for 10 ns with a time step of 2 fs at 1 atm, using the Berendsen barostat. During the production run, the bonds which involve hydrogens were frozen with the SHAKE algorithm [45]. AmberTools12 ptraj [33] was used to compute the RMSDs of the protein, the binding site and the small molecule from the initial restraint release phase to the end of the production simulation, using the first snapshot as reference. The RMSDs were also calculated over the production trajectory, using the average structure of the last 5 ns of simulation as reference.

### 3.4. MM/GBSA Binding Energy Calculations

One of the goals of the MD simulations of the complex was to obtain an ensemble of ligand-receptor configurations over which it is possible to calculate an average binding energy. Similar to our previous work [22], we adopted the MM/GBSA method [46,47] as implemented in the AmberTools12 script *MMPBSA.py* [48]. The binding energy of a ligand-receptor complex was calculated as

(1) $$\Delta G_{bind,\;solv}=\Delta G_{MM,\;vac}+\Delta G_{solv,\;complex}-\left(\Delta G_{solv,\;ligand}+\Delta G_{solv,\;protein}\right)-T\Delta S$$

where $\Delta G_{MM,\;vac}$ is the sum of electrostatic and van der Waals interactions established between the protein and the ligand. The TΔS term models the change in conformational entropy due to the binding, which was neglected in our study. Indeed, we did not require any ranking of the compounds in this case. When using the MM/GBSA method, the solvation terms are given by the equation

(2) $$\Delta G_{solv}=\Delta G_{solv,polar}+\Delta G_{solv,npolar}$$

where the polar contribution of the solvent is calculated solving the Generalized Born equation [45]. All the Δs in the above equations are calculated as the contribution value of the complex minus the values of the ligand and the protein alone, where each contribution is computed as the averaged value over a single MD trajectory where each time the complex is maintained, the protein is removed or the ligand is removed, respectively. The salt concentration was set to 0.15 M. The igb flag was set to 5 [46]. The hydrophobic contribution was calculated as

(3) $$\Delta G_{solv,\;\;npolar}=\gamma \cdot SASA$$

where γ (surface tension) was set to 0.005 kcal/mol/Å^2^, and SASA is the solvent-accessible surface area (SASA) calculated using the linear combinations of pairwise overlaps (LCPO) model method [49]. Per-residue decomposition of the computed binding energies was performed among the residues within 5 Å of the docked pose of CR42-24. We performed all the energy calculations considering the last 5 ns of production MD simulation of the system, using 250 snapshots extracted at regular intervals of 20 ps from the trajectory.

### 3.5. Cell Preparation

All cell lines have been licensed from the American Type Culture Collection (ATCC) Manassas, Virginia (US). Master and working cell banks (MCB and WCB) were prepared by sub-culturing in ATCC-recommended media and freezing according to ATCC recommended protocols (www.atcc.org). Cell line stocks for the assays were prepared from the WCB. The MCB, WCBs and assay stocks were prepared within respectively 3, 6 and 10 passages of the ATCC vial. The complete list of the cell lines used in this study, as well as their genetic status, is reported in Table S1.

### 3.6. Compound Preparation

Solid powders of reference compounds were stored as indicated by supplier. Compounds were weighed on a calibrated balance and dissolved in 100% DMSO. DMSO samples were stored at room temperature. At the day of the experiment, the compound stock was diluted in 3.16-fold steps in 100% DMSO to obtain a 9-point dilution series. This was further diluted 31.6 times in 20 mM sterile Hepes buffer pH 7.4. A volume of 5 µl was transferred to the cells to generate the test concentration range in duplicate. The final DMSO concentration during incubation was 0.4% in all wells. If a compound showed very potent activity, the testing range was expanded to ensure a full dose-response curve could be measured in duplicate. If a compound can only be dissolved in an aqueous solution, the recommended buffer is used instead of 100% DMSO. 

### 3.7. Cell Proliferation Assay

Cells were diluted in the corresponding ATCC recommended medium and dispensed in a 384-well plate, depending on the cell line used, at a density of 200–6400 cells per well in 45 µl medium. For each used cell line, the optimal cell density was used. The margins of the plate were filled with phosphate-buffered saline. Plated cells were incubated in a humidified atmosphere of 5% CO_2_ at 37 °C. After 24 h, 5 µl of compound dilution was added and plates were further incubated. At *t=end*, 24 µl of ATPlite 1Step™ (PerkinElmer, Waltham, MA, USA) solution was added to each well, and subsequently shaken for 2 min. After 10 min of incubation in the dark, the luminescence was recorded on an Envision multimode reader (PerkinElmer). 

### 3.8. Data Analysis

IC_50_ values were calculated by non-linear regression using IDBS XLfit 5. The percentage growth after incubation until *t=end* was calculated as follows:

(4) $$\;{\%}_{growth}=100\%\times \left(\frac{luminescence_{t=end}}{luminescence_{untreated,\;t=end}}\right)$$

This was fitted to the ^10^log compound concentration (*conc*) by a 4‑parameter logistics curve:

(5) $${\%}_{growth,\;fit}=bottom+\left(\frac{top-bottom}{1+10^{\left(logIC_{50}-conc\right)\times hill}}\right)$$

where *hill* is the Hill coefficient, and *bottom* and *top* the asymptotic minimum and maximum cell growth that the compound allows in that assay. 

### 3.9. Curve Fitting

Curves calculated automatically by the software were adjusted manually according to the following protocol: the curve bottom was fixed at 0% when the calculated curve had a bottom below zero. The *hill*was fixed on −6 when the software calculated a lower value. Curves were invalidated when the F-test value for fitting quality was >1.5 or when the compound was inactive (<20% maximal effect), in which cases curves were removed from the graphs. When a curve had a biphasic character, it was fitted on the most potent IC_50_. Incidentally, when technical failures were likely, concentration points were knocked out. This is always shown in the dose-response graphs. The maximal effect (Max effect) was calculated as 100% (signal of untreated cells) minus the curve bottom when the dose-response curve was completely determined for more than 85%. A dose-response curve is considered 100% complete when the data points at the highest concentrations reach the curve bottom. If the completeness was smaller than 85%, Max effect was calculated as 100% minus the average of the lowest signal. In cases where the bottom of the curve was locked on 0%, the maximal effect was always calculated as 100% minus the growth inhibition at the highest concentration.

### 3.10. Cell Genetics

The mutation status of cell lines was established from a combination of public and proprietary data. Based on public data (COSMIC Cancer Genome Project, version 80) [50], we collected mutations, amplifications and deletions in established cancer driver genes that occur in Oncolines™ [51,52]. For further validation, a selection of twenty-three cancer genes were sequenced by nitrogen regulatory protein (NTRC) by targeted and full exome sequencing directly from the cell lines used in Oncolines™. As an extra filter, genetic changes were required to be observed with a preset frequency in patient tumor samples in COSMIC, depending on the type of genetic alteration. This discards sporadic, non-cancer-causing mutations. Cell lines were classified as having a wild type or a mutated genotype, where mutated means: at least one allele changed by point mutation, insertion, deletion, amplification or copy number variation. Analysis was performed on genes that were mutated in at least three different Oncolines^TM^ cell lines (ninety-eight genes in total).

### 3.11. Drug Sensitivity

The relation between measured IC_50_s (calculated as explained in section 3.8) and cell line genetics was determined as follow: a larger subset of the most commonly occurring and best-known cancer genes (thirty-eight in total, plus TLR7) was analyzed with type II ANOVA analysis in the statistical program R. The results were displayed in a volcano plot. For more information about the Oncolines^TM^ methods, refer to [53].

## 4. Conclusions

TLR7 is an endosomal, trans-membrane, homodimeric receptor which is involved in the innate immune response, by initiating the signaling cascade upon binding to PAMPs and DAMPs. Its main activators are viral ssRNA and nucleoside analogues. R-837 (also called imiquimod) was the first small molecule to be FDA-approved for the treatment of gout and skin cancer, in its cream form. R-837 was shown to bind also to the colchicine-binding site of β-tubulin, inhibiting microtubule polymerization in an analogous way to colchicine and its derivatives. Due to this suggested binding site promiscuity, which leads to off-target interactions of R-837, we explored the possibility of CR42-24, a potent colchicine analogue, to bind to the R-837 binding site in TLR7. We employed a series of computational tools to identify and fully characterize the potential binding pose of CR42-24 in such site. Also, we tested the compound in a comprehensive cancer cell line panel, which includes TLR7 mutations among other common cancer mutated genes, to observe the possible TLR7-mediated anti-cancer effect of this compound.

Molecular docking simulations targeting the TLR7 site revealed a binding pose of CR42-24 which was consistent with the R-837 one, showing conserved hydrophobic contact patterns. In addition, the CR42-24 predicted pose showed similarities with the binding pose assumed by colchicine in β-tubulin. In particular, the A and C rings of CR42-24 were involved in van der Waals contacts with two hydrophobic zones within the TLR7 site, in an analogue way of the A and C rings of colchicine in tubulin. MD simulations of the CR42-24-TLR7 complex revealed a stable binding conformation. Also, the averaged binding affinity calculated with the MM/GBSA method for CR42-24 was more favorable than the R-837 one. Finally, an important hydrogen bond between the carbonyl group of the C ring of CR42-24 and residue bK432 was identified by decomposing the free energy of binding between the pocket residues. Regarding the testing in cancer cell lines, we observed a considerable negative IC_50_ shift in terms of cell growth, in cancer cells carrying the TLR7 mutated gene, when compared to wild type cell lines. This suggests a possible interaction between CR42-24 and TLR7, and a TLR7-mediated anti-cancer effect of the compound.

In conclusion, we fully characterized the possible binding mode of CR42-24 to the TLR7’s R-837 binding site through a sophisticated computational workflow. Taking into account the following factors: 1) the similarity between the predicted binding mode of CR42-24 and the R-837 one, which was assessed experimentally, 2) the similarities between the CR42-24 pose in TLR7 and the colchicine pose in tubulin and 3) the negative IC_50_ shift observed for TLR7-mutated cell lines, we suggest a possible off-target interaction between our colchicine derivative, CR42-24, and the TLR7 dimer, with a possible anti-cancer effect. This result is interesting not only to extend the known off-target interactions of colchicine derivatives and provide a potential mechanistic elucidation of immune system suppression by these compounds, but also to develop novel TLR7-targeting small molecules based on the CR42-24 scaffold. The results of the biological assay used in this study were not conclusive in terms of identifying the effect of CR42-24 to TLR7 as agonistic or inhibitory. However, the similarity between the binding poses of CR42-24 and R-837 may suggest a similar activating effect upon binding to the receptor. In addition, R-837 is known to inhibit cell growth especially in skin cancer [54], and we observed a higher sensitivity of TLR7-mutated cell lines to the CR42-24 compound. Future works will include more detailed biochemical and biological testing to assess the direct binding of CR42-24 to TLR7 and the effect that this has on the receptor, i.e., if CR42-24 has an activating or inhibiting effect to the signaling process initiated by TLR7 upon binding to it.

## Figures and Tables

**Table 1:** Results from the MM/GBSA calculations over the last 5 ns of MD production trajectories of the R-837-TLR7 and CR42-24-TLR7 complexes. The total binding energies of the two compounds are in a comparable range, with the more favorable van der Waals and electrostatic contributions of the CR42-24-TLR7 complex being partially compensated by a larger, unfavorable polar desolvation term, when compared to R-837. The total values reported here do not include the entropic terms. The value of the standard deviation is reported in brackets for each term.

	MM/GBSA ΔG (kcal/mol)				
**Compound**	Total	Van der Waals	Electrostatic	Polar solvation	Non-polar solvation
**R-837**	−31.081 (2.531)	−35.028 (2.600)	−18.251 (2.912)	27.331 (2.653)	−5.133 (0.216)
**CR42-24**	−36.055 (3.090)	−46.841 (2.719)	−24.050 (5.273)	41.089 (5.105)	−6.252 (0.239)

## Supplementary materials

Figure S1: The additional four poses of CR42-24 obtained with molecular docking to the R-837 binding site of TLR7. Figure S2: Superposition of the crystallographic pose of DAMA-colchicine in tubulin from pdb ID 1SA0 and the CR42-24 docking pose in TLR7. Figure S3: RMSD trends for the MD production simulation, calculated using the average structure of the last 5 ns as reference. Figure S4: Salt bridge established between aD555 and bK432, keeping the former far from the amine of CR42-24. Table S1: List of the cell lines in the Oncolines™ panel and their genetic status.
