# Supplementary material for: A Novel Interaction Between the TLR7 and a Colchicine Derivative Revealed Through a Computational and Experimental Study
Source: Pharmaceuticals (Basel). 2018 Feb 13;11(1):22. doi: 10.3390/ph11010022 (PMC5874718; doi:10.3390/ph11010022)
Supplement: Supplementary file 1 [file pharmaceuticals-11-00022-s001.pdf]

Supplementary Materials

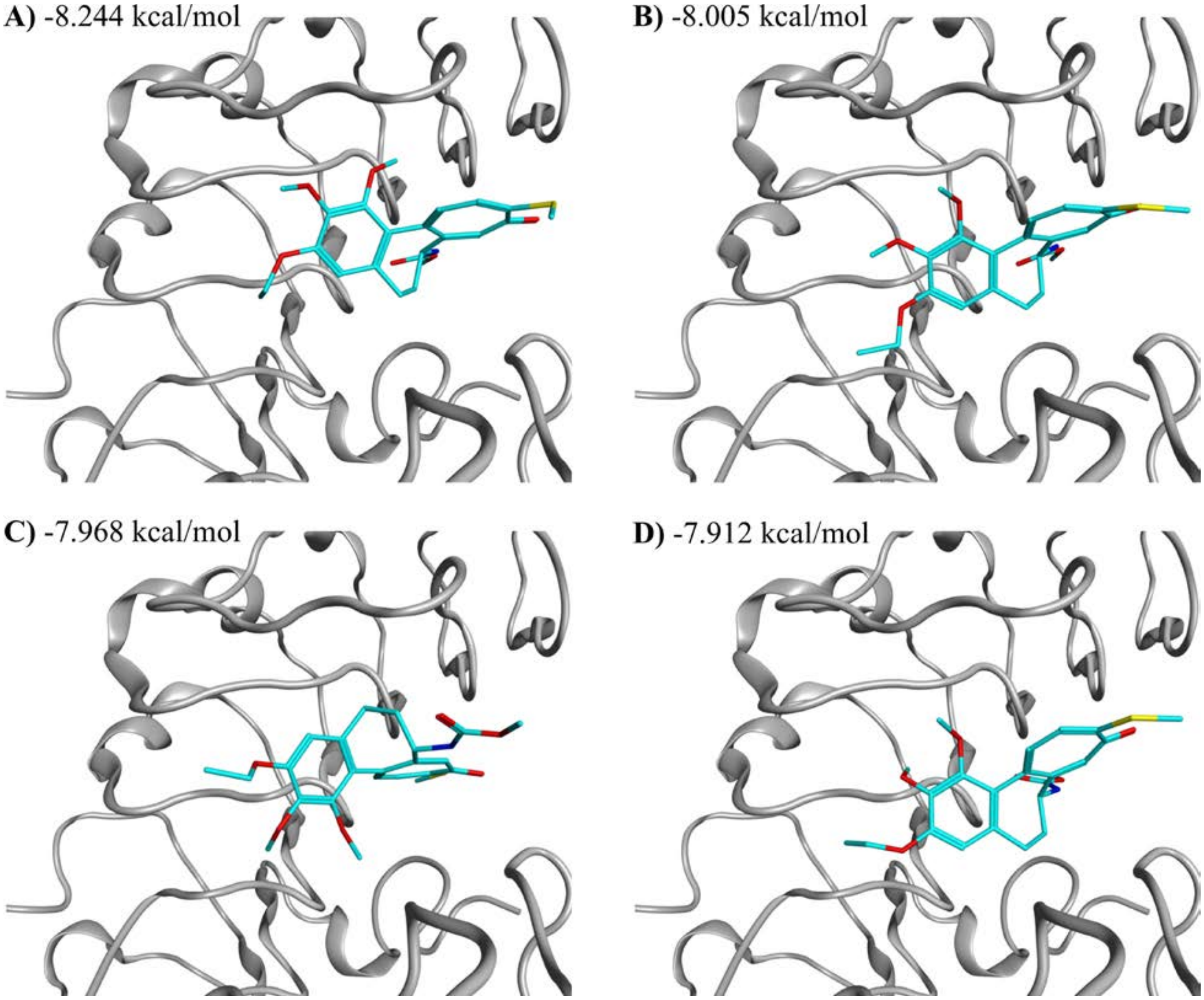

Figure S1: The additional four poses of CR42-24 obtained with molecular docking to the R-837 binding site of TLR7. Computed binding energies are also reported.

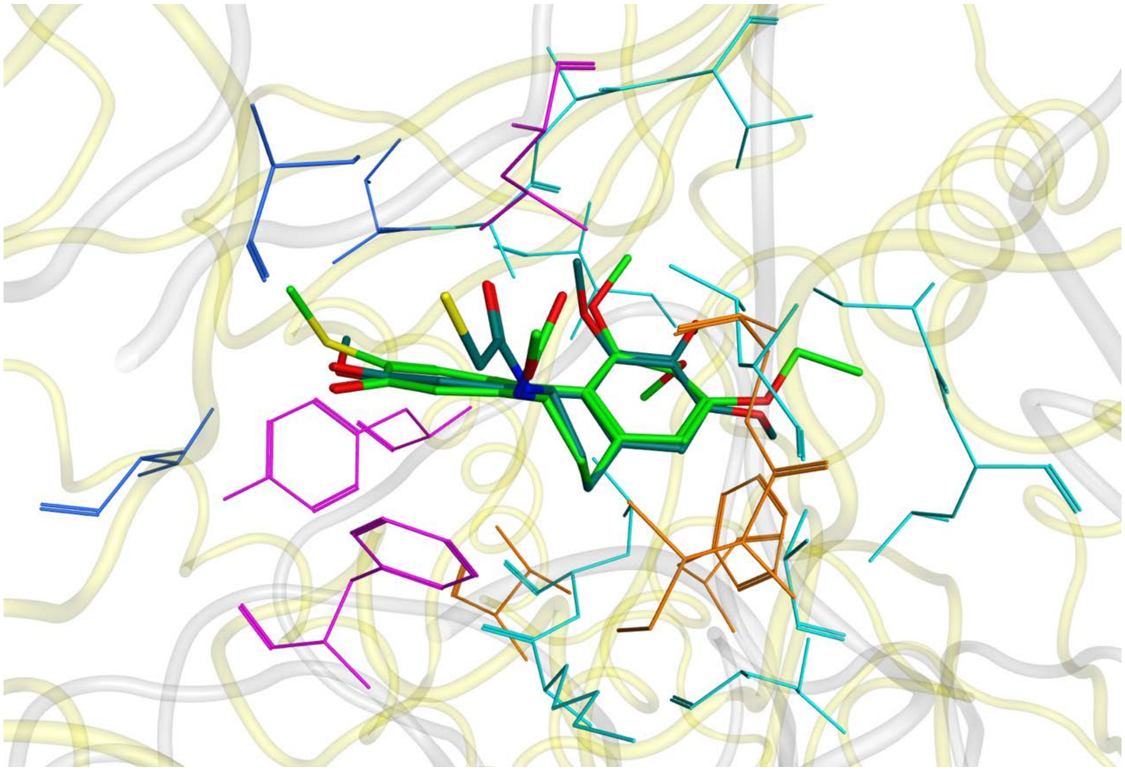

Figure S2: Superposition of the crystallographic pose of DAMA-colchicine (dark green carbons) in tubulin from pdb ID 1SA0 and the CR42-24 docking pose in TLR7 (carbons in light green). The first hydrophobic cleft of the tubulin binding site, interacting with the C ring of the DAMA-colchicine, is reported in blue sticks. The corresponding first hydrophobic zone of TLR7 is reported in purple sticks. The second hydrophobic zone is reported in cyan sticks for tubulin and orange sticks for TLR7. Tubulin backbone is reported in yellow ribbons, TLR7 backbone in grey ribbons.

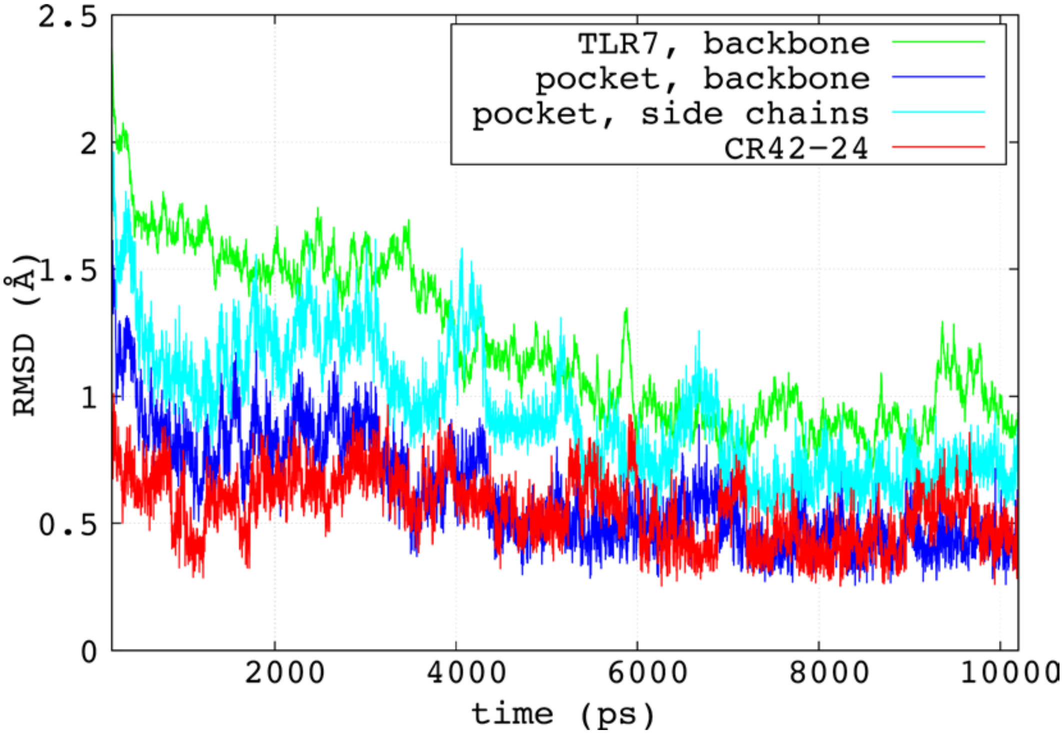

Figure S3: RMSD trends of the MD production simulation for the TLR7 backbone atoms (green), the backbone atoms of the binding pocket (blue), the side chain atoms of the binding pocket (cyan) and the heavy atoms of CR42-24 (red), calculated using the average structure of the last 5 ns as reference. The highest fluctuations are observed for the side chain atoms of the binding pocket, as they adapted to the bound conformation upon ligand binding.

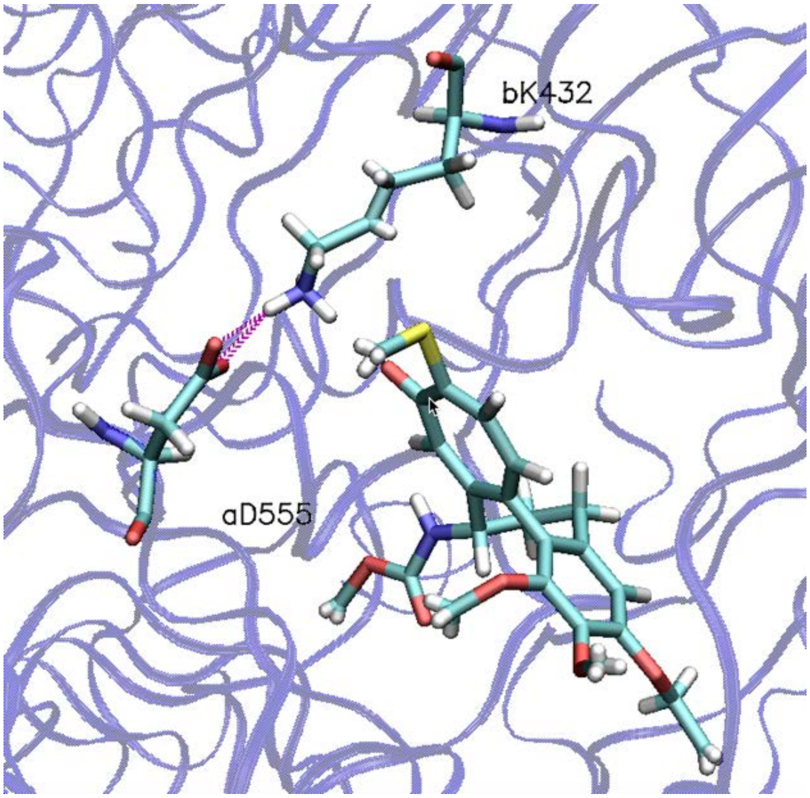

Figure S4: Salt bridge (in purple) established between aD555 and bK432, keeping the former far from the amine of the acetamide group of CR42-24.

Table S1: Cell lines in the Oncolines™ panel used in this study and their genetic status. Indicated in the second column are the genes that are either mutated, part of a translocation or that have altered copy numbers (italic) in that cell line.

Cell line	Cancer genes
5637	ERBB2, RB1, TP53
769-P	No coding alterations found in 38 cancer driver genes
786-O	*CDKN2A*, PTEN, TP53
A-172	*CDKN2A*, *PTEN*
A-204	ABL-driven
A-427	*CDKN2A*, CTNNB1, KRAS
A-498	*CDKN2A*, SETD2
A-549	*CDKN2A*, KRAS, SMARCA4, STK11
A-704	PBRM1, TP53
A375	BRAF, CDKN2A, *CDKN2A*
A388	*EGFR*, NOTCH1, PIK3CA, TP53
ACHN	*CDKN2A*, PBRM1
AN3 CA	CCND1, FBXW7, NSD1, PIK3R1, PTEN, SETD2, TLR7, TP53, ZFHX3
AsPC-1	CDKN2A, FBXW7, KRAS, TP53
AU-565	*ERBB2*, *MYC*, SMAD4, TLR7, TP53

BT-20	*CDKN2A, EGFR*, PIK3CA, RB1, SPEN, TP53
BT-549	PTEN, *RB1*
BxPC-3	BRAF, *CDKN2A*, EP300, *SMAD4*, TP53
C-33 A	ARID1A, FBXW7, PIK3CA, PTEN, SMARCA4, TP53, ZFHX3
CAL 27	CDKN2A, SMAD4, TP53
CCF-STTG1	PTEN
CCRF-CEM	CCND1, *CDKN2A*, FBXW7, KRAS, NRAS, NSD1, *PTEN*, TLR7, TP53
COLO 205	APC, BRAF, *SMAD4*
COLO 829	BRAF, CDKN2A, *PTEN*
Daoy	*CDKN2A, NF1*, PIK3R1, TP53
DB	TP53
DLD-1	APC, EP300, KRAS, NCOR1, PIK3CA, SMARCA4, TP53
DoTc2 4510	BRCA2, FAT1, NSD1
DU 145	CDKN2A, CREBBP, FAT1, *KRAS*, RB1, STK11, XIRP2
DU4475	APC, BRAF, *RB1*
ES-2	BRAF, *CCND1*, TP53
FaDu	*CCND1*, FAT1, *SMAD4*, TP53
G-361	BRAF, *CDKN2A*, STK11
HCT-15	APC, BRCA2, EP300, KRAS, NCOR1, PIK3CA, TP53, ZFHX3
HCT 116	BRCA2, CDKN2A, CHD4, CTNNB1, EP300, KRAS, NCOR1, NF1, PIK3CA, SMARCA4, XIRP2
HL-60	CDKN2A, *MYC*, NRAS
Hs 578T	*MYC*, PIK3R1, TP53
Hs 746T	TP53
Hs 766T	ARID1A, KRAS, *LRP1B*, *SMAD4*
HT	EP300, SPEN, TP53
HT-1080	*CDKN2A*, NRAS
HuTu 80	CTNNB1, SMARCA4
J82	ERBB2, *PTEN*
JAR	No coding alterations found in 38 cancer driver genes
Jurkat E6.1	APC, ARID1A, *CDKN2A*, CREBBP, FAT1, FBXW7, PTEN, SMARCA4, TP53
K-562	ABL-driven, *CDKN2A*, TP53
KATO III	NOTCH1
KG-1	No coding alterations found in 38 cancer driver genes
KLE	*CCNE1*, FBXW7, *KRAS*, TP53
KU812	ABL-driven, TP53
LNCaP FGC	ATM, BRCA2, PIK3R1, PTEN, SETD2, XIRP2
LoVo	APC, ARID1A, FBXW7, KRAS, SPEN
LS 174T	ARID1A, CTNNB1, KRAS, PBRM1, PIK3CA, TLR7, ZFHX3
LS411N	APC, ARID1A, BRAF, EP300, FBXW7, PTEN, TP53, XIRP2
MCF7	*CDKN2A*, EP300, PIK3CA
MeWo	CDKN2A, NF1, TP53, XIRP2

MG-63	*CDKN2A*, *MYC*
MIA PaCa-2	*CDKN2A*, KRAS, TP53
MOLT-4	*CDKN2A*, CHD4, EP300, NOTCH1, NRAS, PTEN, SMARCA4, TP53
NCCIT	PTEN, TP53
NCI-H460	ARID1A, *CDKN2A*, KRAS, *LRP1B*, *MYC*, PIK3CA, STK11
NCI-H661	*CCNE1*, TP53
NCI-H82	*MYC*
OVCAR-3	*CCNE1*, *LRP1B*, TP53
PA-1	NRAS
PC-3	*MYC*, *PTEN*, TP53
PFSK-1	TP53
RD	NF1, NRAS, TP53
RKO	ARID1A, BRAF, BRCA2, EP300, FAT1, NCOR1, NF1, NSD1, PIK3CA, ZFHX3
RL	EP300
RL95-2	ARID1A, ATM, BRCA2, EP300, PIK3R1, PTEN, SMARCA4, SPEN, TP53, ZFHX3
RPMI-7951	BRAF, *PTEN*, TP53
RS4-11	*CDKN2A*, SMARCA4
RT4	*CDKN2A*, *SMAD4*
SHP-77	KRAS, TP53
SJCRH30	TP53
SK-N-AS	NRAS, TP53, XIRP2
SK-N-FI	*NF1*, TP53
SNU-5	*ARID1A*, CDKN2A
SNU-C2B	ERBB2, KRAS, SMARCA4, TP53
SR	*CDKN2A*, ZFHX3
SU-DHL-1	CREBBP, TP53
SU-DHL-6	CREBBP, EP300, TP53
SUP-T1	*CDKN2A*, CHD4, PBRM1, PIK3CA, TP53
SW48	BRCA2, CTNNB1, EGFR, EP300, FBXW7, NCOR1, SETD2, ZFHX3
SW480	APC, KRAS, TP53
SW620	APC, EP300, KRAS, *KRAS*, *MYC*, TP53
SW626	APC, KRAS, SMAD4
SW837	APC, FBXW7, KRAS, TP53
SW872	BRAF, CDKN2A, *PTEN*
SW900	*CDKN2A*, FAT1, KRAS, *KRAS*, *NF1*, *NSD1*, TP53
SW948	APC, ATM, KRAS, PIK3CA, SMAD4, TP53
SW982	BRAF, *CDKN2A*
T24	EP300, FAT1, TP53
T98G	*CDNK2A*, TP53
TCCSUP	ARID1A, PIK3CA, *RB1*, TP53
THP-1	ARID1A, *CDKN2A*, NRAS, *PTEN*, TP53

TT	TP53
U-118 MG	*CDNK2A*, TP53
U-2 OS	*LRP1B*
U-87 MG	*CDNK2A*, NF1
VA-ES-BJ	*CDNK2A*
